# Healthcare Experiences of African American Women with the Fragile X Premutation

**DOI:** 10.1007/s40615-023-01792-2

**Published:** 2023-09-15

**Authors:** Andrew P. King, Nadia Ali, Cecelia Bellcross, Fabienne Ehivet, Heather S. Hipp, Jessica Vaughn, Emily G. Allen

**Affiliations:** 1 Department of Human Genetics, Emory University School of Medicine, Atlanta, GA, USA; 2 Department of Human Genetics, Emory Healthcare, Winship Cancer Institute, Emory University School of Medicine, Atlanta, GA, USA; 3 Division of Reproductive Endocrinology and Infertility, Department of Gynecology and Obstetrics, Emory University School of Medicine, Atlanta, GA, USA; 4 GeneScreen Counseling, Morristown, NJ, USA

**Keywords:** Fragile X, Premutation, FXPOI, African American, Women, Race

## Abstract

This study aims to understand the healthcare experiences of African American women with a fragile X premutation (PM). PM carriers are at risk for fragile X-associated conditions, including primary ovarian insufficiency (FXPOI) and neuropsychiatric disorders (FXAND). There is no racial/ethnic association with carrying a PM, but African American women historically experience barriers receiving quality healthcare in the USA. Obstacles to care may increase mental health conditions like anxiety and depression. Eight African American women with a PM were interviewed to explore disparities in receiving healthcare and to learn about psychosocial experiences during and after their diagnoses. Interviews were transcribed verbatim and independently coded by two researchers. A deductive-inductive approach was used, followed by thematic analysis to determine prominent themes. The average participant age was 52.3 ± 8.60 years, with a mean age at premutation diagnosis of 31 ± 5.95 years. Seven participants had children with FXS. Themes from interviews included healthcare experiences, family dynamics, and emotional/mental health after their diagnosis. Participants reported concerns about not being taken seriously by providers and mistrust of the medical institutions. Within families, participants reported denial, insensitivity, and isolation. Participants reported a high incidence of anxiety and depression. Both are symptoms of FXAND and stresses of systemic racism and sexism. The reported family dynamics around the news of a genetic diagnosis stand apart from other racial cohorts in fragile X research: interventions like family counseling sessions and inclusive support opportunities from national organizations could ease the impacts of a PM for African American women.

## Introduction

Individuals who have a CGG-trinucleotide expansion in the 5’ untranslated region of the *FMR1* gene are at risk for various multi-system health conditions [1]. In X-linked gene, people with a full mutation allele (>200 repeats) are affected with fragile X syndrome (FXS), the most common monogenic cause for autism spectrum disorder (ASD) and intellectual and developmental disability (IDD). Premutation (PM) carriers (55–200 repeats) may experience various other FX-associated conditions. Twenty percent of females who carry a PM experience fragile X-associated primary ovarian insufficiency (FXPOI), which is defined as absent or very irregular menses and menopausal levels of FSH before age 40 [2]. FXPOI can also put women at increased risk for cardiovascular disease and reduced bone density [3]. Males who carry a PM, as well as some women, can develop fragile X-associated tremor ataxia syndrome (FXTAS), with a late-onset neurodegenerative presentation. Most recently, a group of related mental health conditions, including anxiety and depression, have been correlated with a PM and referred to as fragile X-associated neuropsychiatric disorders (FXAND) [4]. The constellation of symptoms of FXPOI, FXTAS, and FXAND often overlap for women who carry a PM [4]. Allen et al. recently found that the diagnosis of FXPOI was associated with anxiety in women that carried a PM, and that symptoms of FXTAS in these women were associated with depression [4]. *FMR1* expansion also leads to a multi-generational expression of the above health conditions, with the potential of a woman carrying a PM having both a father diagnosed with FXTAS in his 60s and a child with FXS from birth, as illustrated by the pedigree from Tassanakijpanich, Hagerman, and Worachotekamjorn [5]. A meta-analysis of prevalence estimates reported 1 in 291 women carry a PM [6]. The majority of women who carry a PM find out through family history, including having a child affected with FXS. Prior research into the diagnostic experiences and health education of women in general, irrespective of race, who carry a PM has shown numerous barriers to the receipt of appropriate healthcare [7, 8]. In clinical settings, many women reported negative experiences with uninformed healthcare providers, fears of not being taken seriously by doctors, and a need to provide their clinician with educational materials about FXPOI [8]. Beyond the clinic, women reported lack of support from family and friends, isolation, and the need to be their own advocates and educators for their needs [7, 8].

Research into the prevalence of a PM in women does not indicate segregation along racial or ethnic lines, yet women of color have been underrepresented in the research around FXS-associated conditions [9]. Visootsak et al. explored the experiences of African American mothers seeking diagnoses for their children affected by FXS [10]. Participants shared delays in children’s diagnoses due to lack of awareness by their family physicians, requiring mothers to educate themselves about FXS and to assertively request testing for their children. After receiving a diagnosis, African American mothers were commonly met with denial and lack of support from family members when sharing the news. These reactions led to decreased cascade testing of potential carriers and strained relationships within families [10].

While Visootsak’s study remains one of the only studies to investigate the experiences of African American women whose children received a FXS diagnosis, further research into the healthcare experiences of the women themselves as PM carriers is warranted. Particularly, as disparities in healthcare access and quality in America have been demonstrated along racial lines, African American women are disproportionately impacted in reproductive and women’s health clinics [11, 12]. There are known psychological, social, cultural, and structural factors that act as barriers to African American women receiving appropriate healthcare. Barriers range from poor health insurance coverage and fewer local medical clinics to providers’ racial biases and patients’ historically founded fears of medical abuse [12, 13]. A relevant schema of coping mechanisms, the “Strong Black Woman” (SBW) schema, has been put forward to explain how individual African American women adapt to these barriers while maintaining dignity. The SBW is “characterized by beliefs such as dedication to the care of others, resilience, ethnic pride, perceived obligation to embrace multiple roles, determination to succeed, and the perceived obligation to exude strength and suppress emotions” [12]. Internalizing this psychological schema may increase the incidence of depression and anxiety for African American women, as the SBW model has been tied to delayed self-care, decreased psychological openness, and increased stress levels [12, 14].

Given the known disparities in healthcare access and the described barriers that people of color encounter, this study aims to explore the healthcare experiences of African American women who carry a PM in more depth, using semi-structured interviews. The study team hypothesizes participant encounters will highlight particular disparities to access and quality of medical services, as well as specific psychosocial experiences African American women face within both their racial community and the network of fragile X support.

## Methods

### Participants

To identify African American women who carry a PM, a list of potential participants was collected through purposive sampling of the Emory Fragile X Center registry, as well as the Fragile X Research Participant Registry of the Carolina Institute for Developmental Disabilities (CIDD). The Emory Fragile X Center recruits from many national sources, including the Emory Fragile X Clinic, Emory Genetics Clinic, Fragile X Clinic and Research Consortium, fragile X family conferences, fragile X listservs, parent support groups, and word of mouth. The Fragile X Research Participant Registry at the CIDD is a national collaboration between the University of North Carolina, Chapel Hill (UNC) and the Waisman Center at the University of Wisconsin which recruits from local, regional, and national fragile X events, as well as through an online registration form. From these sources, 24 potential participants from nine states were emailed an informational flyer about the study, including the recruitment process.

Of the 24 individuals who identified as African American and female contacted by email or phone call, 10 consented (41.67%) to participate in the study. Of those who consented, eight scheduled a qualitative phone interview, while two were lost to follow-up. Detailed demographic data is included in Table 1. Participants received their PM diagnosis between 5 and 28 years ago, with an average of 20.25 ± 6.81 years prior to their interview participation. Their mean age of diagnosis was 31 ± 5.95 years. At the time of the interviews, the mean participant age was 52.3 ± 8.60 years.

### Materials

An interview guide (included in supplementary materials) was developed from a literature review of similar research [8, 10] to explore healthcare experiences, the journey to genetic testing, the disclosure of a participant’s PM results, sources of support and follow-up care, changes of emotional health, and advice from the study participants. It was reviewed by two genetic counselors, a health psychologist, a reproductive endocrinologist, and the primary research investigator. Two pilot interviews were conducted with the genetic counselors (FE and JV), who share racial/ethnic identities with the participants, to improve the flow of questions and ensure appropriate cultural humility.

The interview guide included questions about health conditions participants have which may be associated with a PM, in order to learn more about the care the women were receiving. There was no constraint around the timeframe of these health concerns, allowing participants to share about both prior and developing diagnoses which could impact their health care experiences. Table 2 includes the categories of conditions they were asked about, as well as how many participants reported each condition. All participants reported at least one health condition (range: 1–5), with four participants reporting one or two conditions and four reporting three or more. Mental health diagnoses were the conditions reported most often. Anxiety and depression were the most commonly diagnosed mental health issues, with two reporting anxiety, one reporting depression, and three reporting both.

### Study Design

Eligible participants were emailed the link to an online consent form in REDCap, along with a flyer including information about the study. Prospective participants who responded through REDCap or email were then scheduled for a phone call to complete the consent discussion and an in-depth interview. A genetic counseling Master’s level graduate student (AK) conducted all interviews between May and December of 2022. Individuals who completed the interview were compensated with a $25 Visa gift card.

### Data Analysis

The interviews ranged from 20 to 80 min, were audio-recorded, and sent to a HIPAA-compliant third-party transcription service. De-identified verbatim transcriptions were independently coded using MAXQDA Analytics Pro 2022 by two coders (AK and EA) using deductive codes from literature review and inductive codes arising from the data. Inter-rater reliability (IRR) was calculated using Mezzich’s modified kappa formula for a maximum kappa value of 0.64. Any discrepancies in coding were resolved through discussion after each interview was coded. Coded data were compared for context, analyzed for intersecting codes, and grouped into topics. Thick descriptions of these topics were composed and thematic content analysis was used to organize patterns emerging from the data [15]. Descriptive statistics were calculated using Microsoft Excel 2019 (v. 2302) with Analysis ToolPak add-in. This study was reviewed and approved by the Emory University Institutional Review Board (IRB00045808).

## Results

Four primary themes emerged during data analysis along with relevant subthemes. The primary themes include (1) healthcare experiences in care of PM-associated conditions, (2) family dynamics related to diagnosis of fragile X, (3) mental and emotional health, and (4) advice for healthcare providers, national patient and research groups, and other African American women who carry a PM.

### Theme 1: Healthcare Experiences

The broad theme of healthcare experiences explored participants’ interactions with providers to get care for PM-associated conditions and how accessible care was. Subthemes in this category were: perceptions of health care provider support and knowledge, external barriers to care, and internal barriers to care.

#### Perceptions of Healthcare Providers (HCPs)

##### Supportive/Informed HCPs

Participants described various levels of support from healthcare providers when receiving their fragile X PM care. They reported supportive providers at Fragile X Research Centers, OBGYN practices, and family medicine/primary care practices. Geneticists and reproductive endocrinologists (REIs) were identified as the most commonly informed about a PM. Providers who showed humility about their own knowledge and made appropriate referrals were seen as supportive.

“But never…felt like I wasn’t taken serious. They just kinda like, “Well, that’s not one of the things that typically happen…maybe you should see, you know, someone…at the Fragile X clinic.”

Healthcare providers who educated themselves about the Fragile X PM and collaborated with participants were also seen as supportive.

“I have no issues, and when I do my…PC [primary care]… She’s been able to educate herself… And so anything I need…she’ll say okay—she’ll ask me why of course. And then…if it’s something that she doesn’t think, of course she’s gonna talk to me. But she doesn’t just say no. We have a conversation.”

It was clear that the level of empathy from a provider did not always correlate with their perceived level of knowledge. One participant was referred to a well-informed specialist in the process of receiving her FXPOI diagnosis.

“He, uh, despite the fact that he was a little, you know, kinda dry in the mouth about it, he—what I appreciated was he was very detailed with the explanation…It was traumatizing because it was very definitive…but what I did appreciate is that he explained to me what it was. And when he started explaining that, a buncha other stuff started to make sense. I said, “Oh. Well, then that’s why this was happenin’. And this is why.” You know, um, uh, yeah. It started to make sense. And then when it—when he talked about—I mean, he—I still have the drawing to that when he explained it. And he did the X’s. And then I’m fascinated with science, so I was sittin’ there goin’, “Really?”…He was being very scientific.”

The same participant found her OBGYN offered an empathetic balance to her specialist’s informative consult.

“My main GYN was so concerned, compassionate, all of that. It kinda balanced it out. So I got the information that I needed, and that was helpful. Because my GYN had no idea why that was happening.”

##### Unsupportive/Uninformed HCPs

All participants in this study reported encounters with healthcare providers who were unfamiliar with the fragile X PM. Except for one participant, the cohort members also reported being the one to explain to medical professionals about a PM.

“Doctors for me…have never heard of fragile X or if they have, they have very cursory knowledge of it. And they just kind of accept whatever I tell them…”

A doctor’s reception to learning about fragile X and associated conditions sometimes led participants to find new providers for themselves or their children.

“The doctor was not open to understanding fragile X. He was an older person, and I was like, okay, you can’t learn new things. It’s an old dog— who didn’t wanna know new tricks.”

Other participants perceived moderately informed doctors as less supportive; for example, one participant was trying to plan her family and her provider had some knowledge about FXPOI, but overestimated its penetrance.

“I wasn’t expecting to have any other children actually after him because of—I knew that we had this genetic fragile X in our family, so I had not planned to have any more children. But [laughter]—which, ironically, my doctor told me that, “You know, you don’t have to worry about that. You’re not probably gonna have any more. You’re probably gonna go through early menopause.” And then, sure enough, I ended up pregnant and—- the doctor, you know, he just said, “Well, you know…there’s things in your body evidently still…functioning.”

One participant recounted her daughter’s fragile X evaluation through a national research center which was particularly cold, though the doctor involved was well informed about the condition.

“I do believe it was, like, a clinical…geneticist. Like they were very, I don’t wanna say calculated, but it was very technical. Like it was very…“Okay, we’re gonna run this”…and then he came back in and that’s when they started doin’…the measurements and, like…looking at her and…all that stuff.”

Two participants also shared that they sought out female healthcare providers, in order to receive a quality of care they did not experience with male providers.

“[The endocrinologist] I got changed to, um, he just didn’t listen. I think there’s a lot of stuff that women—you know, nothing against men or anything like that. That, like, you try to…explain and just kinda either gets…brushed off if you don’t understand the experience…that type of thing. So I actually got to the point—like I said, I called…endocrinologists. Like I need a change…or I’m gonna go elsewhere. Like, I’m gonna come out of your practice and go to a different one…and we finally got that ironed out.”

#### External Barriers to Care

Aside from their perceptions about their providers’ knowledge and support, participants reported a variety of barriers to receiving quality healthcare. These were both external, such as insurance problems and providers leaving practices, and internal, such as personal worry over being taken seriously and fear/mistrust of the medical institution.

Four participants reported issues with their insurance as barriers to receiving quality healthcare.

“After getting the diagnosis, me and my husband wanted to try to have another child, but we wanted to try to do some testing before we had the child. And this wasn’t really the doctor’s office, ‘cause they were willing. It was more or less managed care. You know, the insurance. And so I had to write a letter to kind of shake ‘em up and say, okay, guys, you don’t know what you’re doing.”

Four participants also reported issues with continued care when a provider left the practice they used.

“I keep gettin’ new primary cares because they leave the practice and stuff. So I have to wind up—like, right now, I have to find another primary care doctor ‘cause they keep—they leave.”

One participant had a particularly difficult time receiving hormone replacement therapy related to FXPOI after a change in her insurance took her to a different practice, with an uninformed provider.

“The only time I’ve had pushback on that and I fired her, um, at one point …I lost my private insurance ‘cause I divorced my husband and…I have Medicaid now. I had…a doctor in a clinic… Um, I went to this young woman, “I can’t in good… conscience continue to write your… request for meds or your prescription for these hormone replacement. I just don’t believe this.” I said, “Well, this is none of your damn business. This is my body.””

#### Internal Barriers to Care

Half of the participants described incidents in which they were worried about being taken seriously or where they felt mistrust of the medical institution. One participant wasn’t sure if her established providers were listening to her concerns.

“Even just with like the mental health thing, um, was hard to do too ‘cause it was, like, do I really need to talk to somebody—versus I’m just having a bad day or…my primary care was like, “Oh, you’re just havin’ a bad couple months. We can—you know, I can prescribe an antidepressant to you and then, like, antianxiety.” But those made me feel really foggy and just…it didn’t feel like it was working or helping… a little bit of the same with just like some other issues I’ve dealt with health wise. That’s just been like, okay, do people believe me or not? And then just like, I need you to take a look at this.”

For other participants, worry and mistrust affected how they approached seeing a new provider.

“You know, some doctors, they have the God complex…you can’t tell ‘em anything. They know everything. They can do everything…So when I have to go to different doctors where it’s my premutation may come into play, I vet them, so to speak, to see if they are at least knowledgeable of it and if they are able to talk to me as if I am not just an idiot. You don’t have to tell me. You talk to me about my health situation. And I’m not…saying that you don’t know, but you need to talk to me. Even if I’m not as fluent in the medical terminology and maybe I don’t understand but talk to me as if I do understand. I am a human being and somewhat educated.”

### Theme 2: Family Dynamics

The next major theme to emerge from this research was family dynamics. This theme explored the experiences of the participants with their immediate and extended families related to communication around fragile X and roles within their family. The subthemes were: informing families, impact on relationships, and caregiving roles.

#### Informing Families

The majority of the participants had to inform family members of multiple diagnoses: the diagnosis of a child with FXS as well as their own PM diagnosis. Given the close relation of these diagnoses, this research could not separate the communication with and reactions of family members for these items. One participant described a well-received, supportive experience.

“The following year when she was six years old, me and my husband and, of course, <daughter> and my sister and her family, we went to <fragile X research center> to do specific testing, assessments of genealogy type of, um, genetics to find out where did it come from. Who has it? What’s the risk of passing it onto future generations? How can we help <daughter>? Where’s she at cognitively, as well as <nephew>?”

This participant depicted her family as supportive and open to informing other relatives about their diagnoses after receiving group counseling. For seven other participants, however, informing families went less smoothly.

“I do have siblings, and, you know, you’re right, I did talk to my sister about it. But again, she doesn’t really want to hear it, um, because I was concerned that one of her children might also have…a more mild case of Fragile X, but she’s never pursued the testing, so it is what it is.”

“And so I sent out a letter to all of them, this is what’s happened, and you should be aware of it because … I didn’t know if my mom or my dad was—I didn’t know yet. And I didn’t understand how it all worked. I remember my mother saying, once we confirmed it was my dad was a carrier—my mother could be inappropriate sometimes. She sent out a note saying, “It’s not us.””

These experiences were more common throughout the interviews. Participants described a range of reactions that included denial, blaming others, avoiding blame, misattributing symptoms, and lack of support.

“Because obviously if I’m a carrier…it either came from my mom’s side or my dad’s side, you know, and then understand—like tryin’ to walk them through—like, how genetics work —even calling out stuff. My dad was like, “I still don’t feel like it was from me or our side. I feel like it was from her dad’s side.” Me tryin’ to debate, like, “No, that’s not how it works typically. I’m a premutation carrier.””

Two participants had family histories of FXS. For one, the family history was not well communicated to her.

“And I came home and just told…my mom, and then that’s when she said, “Well, I told you, your aunt said…I was like, “No, you didn’t tell me that, and you just told me that, “You know, your aunt’s said there’s somethin’ with the somethin’.”” And I was like, “I can’t take that to a doctor and go, “Well, there’s somethin’ with the boys in the family, in the men—” …You can’t come with half the information. I’m like, “And I’ve been around everybody in the family, and nobody said anything to me.””

For the other participant, whose aunt had two children with FXS, she anticipated more support and understanding from her aunt than she received.

“And I was expecting that she would be—because I knew she was knowledgeable ’cause she had been to conferences—she had done all this research herself. She had two kids, both a male and a female, that she would be this plethora of information and want to share it with me so that I may not face the things that she had faced—dealing with them…Well, when I told her, it was almost like she was happy that somebody else was dealing with what she was dealing with so that everybody else knew that it wasn’t because of what they thought it was…she kinda was excited.”

This aunt’s family had blamed the crack cocaine epidemic of the 1980s and 1990s for her sons’ FXS diagnoses. This participant’s diagnosis allowed her aunt to feel vindicated in refuting that claim.

#### Impact on Relationships

Participants in this study described a number of impacts on relationships after they received diagnoses. Some experienced closeness and support from partners and siblings.

“My husband, he was a wonderful husband. We had been married-been together for 35 years. And, uh, there never was a time that he has not supported, not been there—to do what he needed to do as a father, as a husband. And the same with my family. You know, we are all here to support one another, and if one branch is broken or injured, the whole tree is there to help…”

“I’m very close with [my sister and her children]. I’m always, like, the second parent. My sister lost her husband. Um, and, of course, uh—and we lost our mom. So we’re both here in <City>. And so, you know, we kind of are back and forth between the houses makin’ sure we’re both good.”

Others found their relationships with partners and family strained and broken.

“We don’t live near family anymore ‘cause we—over here in <State>. All my family’s on the East Coast… But it was good and bad. Honestly, I’m kinda glad because…it’s one less thing I have to deal with…their comments or their confusion or their—’cause I got a lotta just, for lack of a better word, ignorant comments…Yeah, in a way it’s kind of a safety net not living near family.”

#### Caregiving Roles

The caregiving roles of these participants often focused on parenting an affected child: addressing their daily living needs, attending to their medical concerns, educating, entertaining, and loving them. Along with traditional roles of childcare, participants took on protective roles for their children, along with advocating for them.

“Um, people described me as a mama bear, and it doesn’t offend me.”

“I’m her advocate—no matter what.”

Beyond parental/childcare roles, participants described having a number of other caretaking roles. One participant helped with her sister’s children as they grew up. Another took care of her aging parents, including her father who was affected with FXTAS. Two participants were both caring for uncles and aunts who are in declining health. Five participants had also had jobs in people-facing careers, which led them to care for many others in their daily life.

### Theme 3: Emotional and Mental Health

The emotional and mental health of participants was discussed in all interviews. This theme explored how participants navigated their diagnoses, sources of support, mental health conditions, and treatment.

#### Emotional Health

Participants endorsed a number of emotional states related to navigating healthcare, receiving diagnoses, and sharing information with their families: trauma, validation, frustration, grief, anger, relief, fear, and guilt.

“Mm. Well, my husband and I cried a lot. Worried about what it might mean.” – reflecting on a PM diagnosis

“The weird thing that really hurt the most, I think, in the results was, “The child most likely got it from their mother,” That hurt ‘cause it was just like, okay, wait a minute. I don’t wanna blame myself, but I feel like it’s kinda my fault.” … reflecting on a daughter’s FXS diagnosis

For another participant, her daughter’s diagnosis offered relief and another level of support.

“And so in a way that was a relief because now I had a direct and a very specific path and diagnosis now that I could use within the school district to get her the proper resources that she needed.”

One participant described the improvement in emotional health she experienced after being prescribed hormone replacement.

“Before I got the hormone replacement therapy it was like being on one side of a window and the whole world’s on the other side. Because you take your feelings of intimacy for granted. And to have no desire whatsoever. And once I started taking those hormones I remember… feeling like a little teeny feeling…I feel like it’s a quality of life issue.”

Six participants reported isolation as part of their emotional journey.

“Mmm, no, heh. I’m alone. I’m on my own, I guess? I’m—it’s just me, you know.”

“Uh, my current husband [is supportive], as much as he can be. I still hold a lot of it in…I’m tired of runnin’ up against the walls and havin’ to, like, justify stuff. So I really don’t tell anybody…or talk to anybody about it. It’s kinda one of those, like, I hate it, but, if I get too emotional, I’ll just, you know, I’ll get in the shower, I’ll cry, kinda get it out.”

“Yeah, I went to one Fragile X convention, I think it was in <City> years ago by myself and I met one Black lady. I’ve never met anybody else Black—that has fragile X.”

Participants found support through different connections, including partners, friends, educators, therapists, and even employers. One participant described the value of her chosen family as a source of emotional care.

“So I had my own family that I kinda created here with close friends that I’ve known since I was 18… though not blood related, but those people accepted me, accepted my son, and so I was still able to create the family that he needed—to be that support while we dealt with all of those things.”

Four participants found support from either in-person or online support groups, though they experienced varied outcomes for themselves and their children.

“So it’s kinda like she has autism, but there’s sort of a reason, right? And- just tryin’ to explain, like, this is why, and she fits in with the autism world in a way, but then in other ways…She has, you know, hypotonia, so she… like, riding a bike, it’s just hard because she has to work so hard to get her muscles to do what… she wants them to do, that she gets frustrated and it’s hard—like, okay, “My kid learned how to ride a bike.” That’s great. Your child has autism- and I get it, but hers [is] that plus muscle things.”

Half of participants mentioned finding support through a church or a religious group. This was highly dependent for each participant, whether they felt like their affected child was accepted in church, or if they felt like they could go on their own.

“But, we sit in the back of the church, just ’cause I feel more comfortable sitting back there with him ’cause he can be, kinda, restless sometimes—so to not be a distraction to others, we just sit in the back of the church, but there’s no special accommodations or anything.”

#### Mental Healthcare

Due to the potential for FXAND, participants were specifically asked about mental health conditions.[4] Six participants endorsed diagnoses of anxiety or depression. One participant endorsed a diagnosis of bipolar disorder (BPD) and another of attention deficit hyperactive disorder (ADHD). Four participants received services from a counselor or a psychiatrist. Four participants felt their symptoms were well-managed with antidepressants or antianxiety medications.

“I have had anxiety all my life, but never knew what it was until I was in school for psychology. …they started talkin’ about what anxiety feels like, and I’m like, “I’ve been feeling that way all my life and never knew that that’s what it was.””

“I think it’s just situational depression related to, you know, just takin’ care of my son on my own for 32 years. And the anxiety, too, my doctor says, “It’s probably all tied into just taking care of him.””

### Theme 4: Advice

#### Advice to Healthcare Providers

All participants were asked what advice they had for healthcare providers to improve their diagnosis and care. They shared the hope that providers would be more empathetic, better informed about the fragile X PM, and to present that information supportively.

“It’s one of the things I do think is important for women of color because, again, we don’t—I suppose we don’t get a lot of good information, and it’s not presented in a way that’s supportive.”

They also discussed the need for appropriate referrals for further care related to PM-associated conditions and, specifically when diagnosed through the diagnosis of a child, that parents receive referrals too. Finally, two participants suggested including genetic conditions on intake material in some way, in order to minimize the number of traumatizing questions they receive in a healthcare appointment.

“One of the questions that should be listed is “Do you have some type of genetic condition?” You know, it took me some years to not be traumatized by it… some people still have kids. It’s just all the other stuff that goes along with it. And learning the information yourself. So imagine somebody that doesn’t have the information. They just know they have a genetic mutation or they have a genetic condition, and they’ve gotta explain to a doctor what’s goin’ on with them. It’s like relivin’ it over and over and over again.”

#### Advice to National Patient and Research Groups

Two participants had advice for national patient and research groups like the National Fragile X Foundation (NFXF) and FRAXA. One piece of advice is to be more inclusive and supportive of single parents raising kids with FXS by not presuming there is another adult caregiver in the home.

“And so I had to raise my hand at the—one of the panels, and I was like, “Okay, you keep givin’ techniques—well, if you’re frustrated, let your spouse come in. And if you get frustrated, tag team.”… It wasn’t a tag team. It was me.”

One participant, who cares for two adult children with FXS, wanted to see more research into the transition into adulthood for those who have a full mutation, not just their pediatric care.

#### Advice to other African American Women with a Premutation

Finally, when asked what advice they would give other African American women with a PM, participants overwhelmingly encouraged them to become self-advocates and to seek out educational materials. They suggested seeking out support, both in person and online, and particularly to seek out support from the African American community. Half of the participants suggested women become involved in research. Finally, participants wanted other women to be sure to take care of themselves.

“Take care of yourself. And talk to your other family members…and communicate with your doctors and have them make sure that they take good care of you.”

## Discussion

This study describes the healthcare experiences of eight African American women with a fragile X PM. While the focus of this research was on healthcare disparities these women may experience, the most salient findings were related to how families received the information of a genetic condition with a multigenerational impact. With one exception, the cohort reported difficult family dynamics, including denial and blame from family members when they were informed. The one participant who described a supportive family dynamic in the “Informing Families” section experienced her diagnosis in a family counseling session at a Fragile X Research Center. The difficulties with family communication reported by the majority of participants were consistent with those noted in Visootsak et al. almost a decade ago, where African American women specifically shared experiences about the diagnosis of their children with FXS [10]. The family dynamics observed in this study were also described by one study of African American families with a son on the autism spectrum [16].

A full investigation into the attitudes and cultural perspectives which led to these family dynamics is beyond the scope of this study. Generational mistrust of the American healthcare system among African Americans is documented and well-founded in historical mistreatments, a mistrust which may be shared to varying degrees within extended family units [12]. The negative family dynamics experienced by these participants are an important area for future research. Directions of such research could explore historical attitudes around disability, the effectiveness of family counseling sessions for genetic conditions, and the ways family support differs between African American families and European/White families.

The present study confirms the prior research of Poteet et al. on the medical care of women with a PM, particularly their concerns regarding being taken seriously by their providers [8]. This study came as a follow-up study to Poteet et al., which lacked racial diversity in its study population. Given the heterogeneity of PM presentations in this cohort, the providers seen for diagnosis differed from those of women interviewed by Poteet et al. The current cohort was overwhelmingly diagnosed due to family histories of FXS or PM-associated conditions, often by the provider who tested their affected children. The cohort in Poteet’s study were all diagnosed specifically with FXPOI due to either symptomatic presentation or a positive family history for PM-associate conditions. Consistent with Poteet’s study, this study’s participants experienced providers who were unfamiliar with fragile X and PM-associated conditions. The most knowledgeable healthcare providers reported by the cohort were also REIs and geneticists [8].

Compared with barriers reported by Poteet’s cohort, this study’s participants reported unique barriers to care that included insurance problems, difficulty finding continuity of care, and a general mistrust of the medical system. Burnout and attrition of minority status providers, including African American physicians, was examined in a 2019 review. The authors noted that since physicians from underrepresented minorities (URM), “are more likely to locate or remain in geographical areas with physician shortages and work with vulnerable patient populations, loss of URM physicians is likely to disproportionately affect minority/vulnerable patients, further increasing healthcare disparities” [17]. Participants in the present study were not asked about the racial/ethnic identities of their providers, but the same review stated that URM physicians cared for over half of minority status patients in America. A survey of physicians considering leaving their practices found that higher percentages of uninsured and Medicaid/Medicare patients, as well as difficulties obtaining specialty services for patients, were both strong factors in the turnover of healthcare providers [18]. Another study has shown American providers made significant changes to clinical management in response to changes in patient health insurance status [19]. Participants in the current study reported similar issues such as physician attrition and changes in their care based on their insurance status as accessibility issues. Other structural disparities described elsewhere (transportation, work-related, and scheduling barriers) were not described by the current study’s participants [20]. This may have been due to the fact that this cohort was made up of educated and professionally employed participants.

Some aspects of the participants’ experiences may track with the “Strong Black Woman” (SBW) schema, laid out by Belgrave and Abrams, particularly methods of showing strength, such as “postponement of self-care and emotional suppression” [12]. Women in the study were asked about their dedication to the care of others and their willingness to embrace multiple roles in their family structure. Participants often provided dedicated care for family members beyond their children and partners. The majority found employment in careers which required them to provide physical or emotional care professionally. More expressive aspects of the schema could not be applied through phone interviews. Future research in this area should be considered by investigators with greater expertise in psychology and cultural anthropology.

This study recorded a high incidence of anxiety and depression among participants. It was not within the design of the study to distinguish between mental health conditions related directly to FXAND, the stresses of caring for a child with an intellectual disability, and those inflicted by intersectional oppression of racism and sexism in America [4, 14]. A quantitative study of the SBW schema and mental health found self-silencing to act as a mediator between coping strength behaviors and depressive symptomatology [14]. Current participants did not report themselves as self-silencing in medical settings. Quite the opposite, many in this study had become strong self-advocates for their care, even when they met barriers. The compounded difficulties of caring for people affected with FXS, as indicated by one participant’s assertion that her 32 years of caring for her child may be the cause of her mental health conditions, complicates the usage of the SBW schema in this population. Other caregiving responsibilities that fall within a fragile X family could also impact mental health conditions, such as caring for an older family member with FXTAS.

Further research, aside from previously mentioned in the areas of family dynamics and expressive elements of the SBW, should evaluate the understanding of healthcare providers, particularly OBGYNs and family practice physicians, about the fragile X PM and supportive means of reaching female patients. NFXF is launching an initiative with two Fragile X Research Centers to survey minority-status stakeholders on their concerns around representation in clinical research and healthcare settings (H. Rosselot, personal communication, March 27, 2023), which should further explore the findings of this study. Studies into the effectiveness of racially-concordant support groups for women with children who have intellectual disabilities should also be considered, as isolation among the participants may have been mitigated by such interventions.

## Limitations

The sample for this study was small and dispersed between the Southeast, Midwest, and the Mountain West. This study did not account for current household income; although, it is clear that the study population is highly educated. Five of the eight women have a Master’s degree, and all participants completed at least some college. Additionally, selection bias may have led to participants’ ability to take part in the study. Over half of these participants had access to a Fragile X Research Center, giving them greater access to resources and information. The racial and gender discordance between the interviewer (AK) and the participants may also have limited the depth of experiences shared throughout the interview process.

## Conclusions

The healthcare experiences of African American women with a FX PM are impacted by the support and knowledge of their providers, as well as their own worries about and trust in the institution of American medicine. Beyond the medical establishment, there is a need to find ways to support women, their children, and their families as they navigate the difficulties of a genetic diagnosis which can have multigenerational impacts. The NFXF is well-situated to hear from African American women and their families to highlight the needs they experience in medicine and advocacy. Collaboration with healthcare providers will be critical to address the unmet needs experienced by the participants in this research. It is clear that many challenges that were identified by Visootsak et al. [10] more than ten years ago still exist in the current study. Given this lack of improvement, a dedicated task force or resources for support groups within NFXF to understand and address the needs of African American families in the fragile X community could serve a critical need. In fact, the current work to establish an International Fragile X Premutation Registry from the NFXF can be used to bring together this population of women and provide these resources. Facilitating genetic counseling sessions with more second degree relatives present may be an effective intervention for some of the family dynamics described by participants. Other interventions by genetic counselors and mental health professionals, along with establishments of community support, should be evaluated for the appropriate care of African American women impacted by fragile X.

## Supplementary Material

Supp Info

## Figures and Tables

**Table 1:** Participant demographic information

	*n* = 8
Mean age (years)	52.3 ± 8.60
Mean age at PM dx (years)	31 ± 5.95
# Children	
0 children	1
1 child	2
2 children	4
3 children	1
# Children w/ FXS dx	
1 child	5
2 children	2
# Participants w/ POI dx	2
# Participants seen by FX Research Center	5
Marital status	
Single	2
Married	1
Divorced	2
Divorced and remarried	2
Widowed	1
Educational degree	
Some college	1
Bachelors	2
Graduate	5

*FXS* fragile X syndrome, *dx* diagnosis, *PM* premutation, *POI* primary ovarian insufficiency

**Table 2:** Possible premutation-associated health conditions

Condition	Number of participants reported (*n* = 8)
Fertility issues	5
Low bone mineral density	3
Ataxia	0
Tremor	2
Neuropathy	3
Mental health diagnosis	6
Hypertension	2
Diabetes	3
Autoimmune disease	0
