# Supplementary material for: Healthcare Experiences of African American Women with the Fragile X Premutation
Source: J Racial Ethn Health Disparities. 2023 Sep 15;11(6):3390–400. doi: 10.1007/s40615-023-01792-2 (PMC11086630; doi:10.1007/s40615-023-01792-2)
Supplement: Supplementary file 1 [file 40615_2023_1792_MOESM1_ESM.docx]

Interview Guide

1. How old are you? 

2. What level of education did you receive? 

3. What is your current occupation? 

a. What did you do, if retired?

4. What is your ethnicity?

5. Do you have any children? 

a. If so, how many? How old is each?

b. Are they your biological children?

6. Are you in a relationship/married? 

a. If married: Is this your first marriage?

7. Who do you take care of in your personal life?

a. Anyone beyond children, partner?

b. What sort of care do you provide these relatives/friends/pets?

8. How old were you when you learned you are a carrier, which means you have the premutation for fragile X syndrome?

a. What led you to get fragile X testing?

b. Who provided you with your testing for your carrier status?

i. Child with FXS?

1. How long did it take to get child diagnosed?

2. Were you told any other reasons for your child’s condition prior to their diagnosis?

ii. Research?

iii. Personal medical care?

c. Were you referred or did you talk to any other medical professionals about your results? Were you referred to a genetic counselor?

9. What has been your experience sharing this information with your family if you have chosen to do so? 

a. Have they been supportive? 

b. Do they understand the information? What resources were helpful?

c. Have they also chosen to receive genetic testing?

**General healthcare questions:**

1. Tell me about your general health?

a. Any particular health conditions? 								*If any conditions not known to participant, suggest seeing GC*

i. Fertility/OB 

1. Are you still having your period? 

2. Have you ever taken hormone replacement therapy?

ii. Heart 

iii. Tremor/Ataxia 

iv. Thyroid/Endocrine 

v. Bone

vi. Fibromyalgia/neuropathy

vii. Autoimmune disease

viii. Mental health 

*If you receive multiple flat answers to topic, drop it.*

b. When did you start having symptoms for [specific condition]?

i. When were you diagnosed with [specific condition]?

c. Have you had any surgeries? 

2. What have your experiences been like receiving care for this/these condition(s)?

a. What type of doctors treat you? 

b. What advice did you receive from doctors when symptoms of [condition] came on? 

c. Do you see any specialists?

d. Could you describe the care you are receiving in relation to your PM? 

3. What challenges have you experienced in the process of receiving healthcare?

a. **If not mentioned prior, probe:**

i. Financial 

ii. Insurance

iii. Transportation

iv. Timing

v. Childcare

vi. Family support

vii. Not a personal priority

viii. Fears/concerns for personal welfare

ix. Not being heard/taken seriously

x. Other reasons we haven’t discussed

b. How would you describe the care you received?

c. How familiar were you with FXS before your found out you were a premutation carrier? Where did you look for information about FXS/PM?

d. Have you ever had to bring educational materials about FXS or the PM to your doctors?

4. Have you received a clinical diagnosis of primary ovarian insufficiency?

a. **If Yes**: 

i. What age were you diagnosed?

ii. How did you receive this diagnosis?

b. What doctor diagnosed it?

i. How much time was there between the onset of your symptoms and your official diagnosis?

ii. How many doctors did you see?

iii. What did doctors say to you at the time of your diagnosis?

iv. Do you remember waiting for your test results? How did you feel?

v. Do you remember the conversation about your results? How did you feel?

c. **If they learned about status from research:** Did you tell any of your providers about your premutation carrier status?

i. Which providers?

ii. Were they familiar with Fragile X premutations and their impact on health and wellbeing?

d. Did finding out you have the premutation affect your decision to have children? If so, how?

5. Did you feel emotionally supported during your premutation diagnosis?

i. **Yes:** What helped you feel supported?

ii. **No:** What made you feel unsupported after your diagnosis?

b. Who has cared for you? 

i. Particular relationships?

ii. Institutions/organizations?

**Advice**

6. Do you have any advice for healthcare providers to improve the experiences you have had with your healthcare? 

7. Do you have any advice for other African American women trying to receive healthcare as a premutaton carrier? 

8. Is there any other information you would like to add before we conclude the interview?
